# Associations between Sleep Hygiene and Mental Complaints in a French Healthcare Worker Population during the COVID-19 Crisis: A Cross-Sectional Analysis to Personalize Sleep Health Interventions

**DOI:** 10.3390/clockssleep6020017

**Published:** 2024-04-22

**Authors:** Julien Coelho, Jean-Arthur Micoulaud-Franchi, Pierre Philip

**Affiliations:** 1 SANPSY, UMR 6033, CNRS, University of Bordeaux, F-33000 Bordeaux, France; jarthur.micoulaud@gmail.com (J.-A.M.-F.); pierre.philip@u-bordeaux.fr (P.P.); 2 CHU Bordeaux, Service Universitaire de Médecine du Sommeil, F-33000 Bordeaux, France

**Keywords:** sleep, social jetlag, healthcare workers, regularity, occupational health

## Abstract

Healthcare workers often have irregular work schedules and experience significant stress, which can lead to poor sleep quality and frequent mental health issues, especially in the context of the COVID-19 pandemic. In this cross-sectional study, we aimed to assess the prevalence of poor sleep hygiene and mental health complaints among healthcare workers and examine their associations. We investigated participants’ typical sleep–wake patterns on workdays and free days as indicators of sleep hygiene. Sleep efficiency and social jetlag were calculated as the ratio of mean sleep duration to time spent in bed, while sleep rebound was defined as the difference in mean sleep duration between workdays and free days. Social jetlag was determined as the difference in mid-sleep timing between workdays and free days, with mid-sleep defined as the midpoint between bedtime and wake-up time. Insomnia severity was assessed using the Insomnia Severity Index (ISI), daytime sleepiness using the Epworth Sleepiness Scale (ESS), and symptoms of anxiety and depression using the Patient Health Questionnaire 4 (PHQ-4). Fatigue was measured using a single item inspired by the Maslach Burnout Inventory (MBI). A total of 1562 participants (80.5% women, mean age 40.0 years) were included in the study. The results revealed that 25.9% of participants slept less than 6 h, 24.3% had a sleep efficiency of less than 85%, 27.3% experienced a sleep rebound of more than 2 h, and 11.5% reported a social jetlag exceeding 2 h. Additionally, 33.9% of participants reported insomnia, 45.1% reported excessive daytime sleepiness, 13.1% reported fatigue, 16.5% reported symptoms of depression, and 35.7% reported symptoms of anxiety. After adjustment, mean sleep duration and sleep efficiency were associated with most mental health complaints. Sleep rebound and social jetlag were associated with significant insomnia but not with anxiety or depression symptoms. Our findings underscore the high prevalence of poor sleep hygiene and mental health complaints among healthcare workers, exacerbated by the COVID-19 crisis. We advocate for the promotion of sleep health through behavioral sleep strategies to safeguard the well-being of healthcare professionals.

## 1. Introduction

Sleep is crucial for overall health and well-being. Insufficient sleep and sleep disorders are highly prevalent and associated with adverse health outcomes [1]. Sleep health is a recent public health concept introduced in 2014 that addresses both insufficient sleep and sleep disorders to respond to this public health issue [2]. It is particularly important in the design and evaluation of behavioral sleep strategies, which could benefit populations at risk, such as hospital workers. Indeed, these professionals have atypical working schedules with frequent night shifts and on-call duties [3]. In addition, they are exposed to experienced stress that leads to loss of sleep and manifested symptoms of sleep deprivation. Mental complaints can affect a physician’s health and also lead to an increased number of medical errors [4]. In 2020, hospital workers have been facing a dramatic pandemic with extreme work pressure, fast adaptations to intense critical care situations, unseen amounts of severe critical patients, numerous deaths of patients, and risks of infection. Lockdowns, social distancing, quarantines, fear about oneself and one’s loved ones, and economic consequences have further increased sleep disruptions in this population [5]. Sleep hygiene is defined as a set of behavioral and environmental recommendations intended to promote sleep health. Research has demonstrated links between individual sleep hygiene components and subsequent sleep [6]. To our knowledge, sleep hygiene has not been evaluated among a French hospital worker population. Assessing sleep hygiene in this population could enable us to propose specific prevention campaigns, improve the overall health of this population and ultimately improve the quality of care. Thus, our study aimed to determine the prevalence of poor sleep hygiene and mental complaints and their associations in a French hospital worker population. By identifying factors associated with poor sleep hygiene and mental health outcomes, we can develop personalized interventions to promote sleep health and improve the overall well-being of healthcare workers.

## 2. Results

Participants included 1562 adults ranging from 18 to 75 years old (40.0 ± 11.2), which represented 12.5% of the 12,495 Bordeaux University Hospital’s professionals. Participants were predominately women (80.5%) and held at least a bachelor’s degree (91.2%). The mean sleep duration was 6 h45 ± 55′ with 25.9% of participants sleeping less than 6 h, while the mean sleep efficiency was 89% ± 9% with 24.3% of participants having less than 85%. A total of 27.3% of participants reported a sleep rebound of more than 2 h and 11.5% reported a social jetlag of more than 2 h. The mean ISI was 11.9 ± 5.6 with 33.9% of the patients reporting significant insomnia and the mean ESS was 10.1 ± 4.7 with 45.1% of the patients reporting significant EDS. A total of 13.1% of participants reported fatigue more than once a week, 16.5% reported significant depression and 35.7% reported significant anxiety (Table 1).

**Figure 1:**
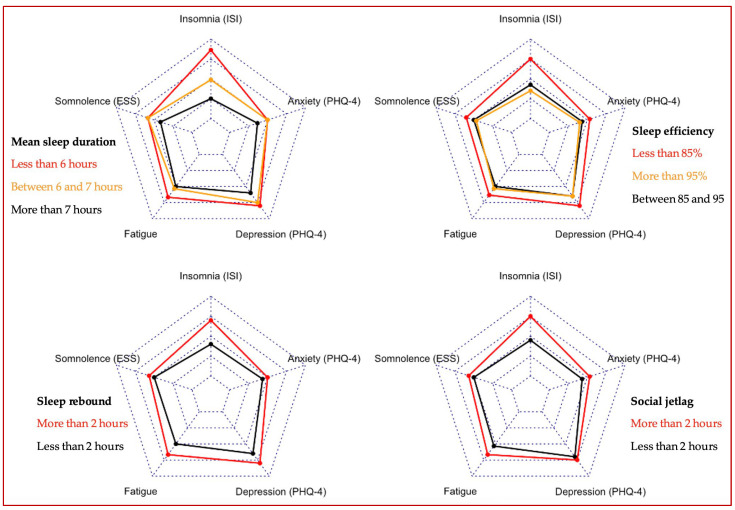
Univariate associations between sleep hygiene and mental complaints (*n* = 1562).

Participants sleeping less than 6 h reported more insomnia and fatigue than those sleeping between 6 and 7 h (Figure 1). Mental complaints were further reduced in those sleeping more than 7 h (mean ISI: 10.0 vs. 11.9 and 14.9, mean fatigue: 3.5 vs. 3.6 and 4.0). Participants with a sleep efficiency less than 85% reported more mental complaints than those with a sleep efficiency between 85% and 95% or those with more than 95%. The results were similar with sleep rebound and social jetlag of more than 2 h.

The results of multivariate logistic regression are presented in Table 2. After adjustment for age, gender, profession, financial situation and work schedules, the mean sleep duration was associated with insomnia (*p* < 0.001), EDS (*p* = 0.031), depression (*p* < 0.001) and anxiety (*p* < 0.001) but not with fatigue (*p* = 0.646). The frequency of insomnia and anxiety were 4-times higher and 2-times higher in participants sleeping less than 6 h compared to participants sleeping more than 7 h, respectively. Sleep efficiency less than 85% was associated with each mental complaint (OR ranging from 1.4 for EDS to 1.9 for insomnia and fatigue). A sleep rebound of more than 2 h was associated with significant insomnia and fatigue (respectively OR = 1.5 [1.2–2.0] *p* = 0.001 and OR = 1.5 [1.1–2.1] *p* = 0.026). Social jetlag was associated with significant insomnia (OR = 1.9 [1.3–2.7] *p* < 0.001) but no other mental complaints.

## 3. Discussion

This study found that poor sleep hygiene and mental complaints were frequent in healthcare workers during the COVID-19 crisis. These results were consistent with two recent meta-analyses. The first published in 2020 found a prevalence of insomnia, anxiety and depression of 34.3%, 23.2% and 22.8% in a population of 33,000 healthcare workers included from 13 studies [7]. The second published in 2021 with 52,000 healthcare workers included from 22 studies found a prevalence of sleep disorders, anxiety and depression of 44.0%, 30.0% and 31.1% [8]. In France, a 2020 study carried out among 1001 young surgeons found that 43.1%, 35.9% and 40.8% of participants had significant insomnia, anxiety and depression, respectively [9]. In our study, mental complaints were less frequent, especially for depression (16.5%), which can be explained by the fact that our questionnaire took place during the summer of 2020 between two epidemic waves. Before the outbreak, a meta-analysis published in 2020 found a sleep disturbance prevalence of 39.2% in a population of 32,000 Chinese healthcare workers [10]. In France, a 2015 study found an average sleep duration (6.5 h) and a prevalence of EDS (44%) equivalent to ours in a population of anesthesiologists and intensivists [11]. These elements show that the COVID-19 crisis worsened an already tense situation for healthcare workers. The deployment of behavioral sleep strategies could therefore be beneficial during the pandemic and even afterwards. Our study also found a fairly low prevalence of social jetlag, which is consistent with studies that showed it improved over this period [12]. Our results showed that sleep efficiency and mean sleep duration were associated with each of the mental complaints except fatigue with mean sleep duration, suggesting that these are the two most important dimensions of sleep hygiene in preventing mental complaints. Social jetlag and sleep rebound were both associated with insomnia but not with EDS, anxiety or depression. These results lead us to consider the importance of the following three behavioral sleep strategies, in this order: (i) not staying awake in bed, (ii) sleeping more than 6 h per night and (iii) keeping regular rhythms on weekends. The challenge now is to promote these messages in partnership with occupational health services, and using validated interventions, with the aim of modifying sleep behavior and ultimately improving the sleep and mental health of these professionals.

Our study has some limitations. First, participants were included on a voluntary basis, so people interested in the issue of sleep were over-represented. However, the sample was representative of the overall population of Bordeaux healthcare workers in terms of age (43.0 vs. 40.0) and gender (80.0% vs. 80.5% of women). Second, the self-administered questionnaire may have led to some misclassification and no objectives measures were used to assess sleep–wake timings. However, we only used validated scales. Also, data are missing regarding usual consumption including tobacco, alcohol, and hypnotic drug intake, which may have influenced sleep and mental health. Further studies should explore how extensive subjective measures and objective measures correlate with each other and with mental complaints. Third, our analyses were only cross-sectional and did not provide information on the direction of the relationship between sleep hygiene and mental complaints. The exact mechanisms underlying these associations remain speculative and should be explored in longitudinal studies. Fourth, it is essential to acknowledge the limitations of our study in assessing the specific contributions of the COVID-19 pandemic to sleep issues among healthcare workers. While our research was conducted during the pandemic, we recognize the need for comparisons with pre-pandemic studies to better understand any changes in sleep patterns over time. Future longitudinal studies may provide valuable insights into the long-term effects of the pandemic on sleep and mental health among the same population.

Our study contributes to the growing body of literature highlighting the significant associations between sleep hygiene and mental health outcomes among healthcare workers, particularly during the COVID-19 crisis. The COVID-19 pandemic has undoubtedly exacerbated existing challenges associated with shift work, such as disrupted sleep patterns and increased stress levels. However, disentangling the specific contributions of shift work and the pandemic to sleep issues and mental health outcomes remains a complex task. Future research endeavors should aim to elucidate these relationships and develop targeted interventions to support the well-being of shift workers, particularly in light of ongoing public health crises. In addition to its implications for healthcare professionals, our study findings may have broader relevance to workers in other industries that also engage in shift work. The challenges associated with shift work, such as disrupted sleep patterns and increased risk of mental health issues, are not unique to the healthcare sector. Therefore, our findings may offer valuable insights for developing interventions to address sleep hygiene and mental health in various occupational settings. However, several research gaps remain to be addressed. While we acknowledge the influence of shift work on sleep patterns and mental health outcomes among hospital workers, the historical practice of long hours for new interns, although gradually changing, remains a concern due to its potential adverse effects on sleep, mental health, and medical errors. Future longitudinal studies may provide further insights into the complex interplay between shift work, the COVID-19 crisis, and their combined effects on sleep and mental health, explore the underlying mechanisms linking sleep hygiene to mental health outcomes in this population, and study the effectiveness of interventions targeting sleep hygiene, in addition to investigating the long-term implications of poor sleep health, including its impact on occupational performance and patient care. Longitudinal studies are warranted to establish the temporal relationships between sleep hygiene, mental health, and occupational outcomes among healthcare professionals.

## 4. Materials and Methods

This study used an observational cross-sectional design conducted in summer 2020. All professionals working at Bordeaux University Hospital were asked to answer an internet-based questionnaire.

Participants were asked about their socio-demographic data (age, gender, profession and financial situation), their work schedules (night and/or shifted) and their COVID-19 exposure (work in a COVID-19 unit).

Concerning sleep hygiene, participants were asked, during workdays and free days, what time they usually go to bed (bedtime) and get up (getting up time) and how many hours of actual sleep they get (sleep duration), based on the methodology proposed by the Munich ChronoType Questionnaire (MCTQ) [13]. From these answers, the following proxies of their sleep hygiene were estimated: mean sleep duration (including workdays and free days), sleep efficiency (ratio of mean sleep duration over time-in-bed), sleep rebound (as the difference between mean sleep duration before workdays and before free days) and social jetlag (as the difference between mid-sleep during workdays and mid-sleep during free days, mid sleep as the middle between bedtime and getting up time [14]). Mean sleep duration was categorized into 3 groups: less than 6 h, between 6 h and 7 h, and more than 7 h, according to the guidelines of the National Sleep Foundation [15]. Sleep efficiency was categorized into 3 groups: less than 85%, between 85% and 95% and more than 95%. Sleep rebound and social jetlag were defined as at least a 2 h shift, according to previous studies [14,16].

Concerning mental complaints, insomnia was assessed with the Insomnia Severity Scale (ISI), a 7-item scale rated on a 5-point Likert scale [17]. We consider a score of 15 or above as moderate or severe insomnia. This scale has shown great reliability and validity in a previous meta-analysis [18]. Excessive daytime sleepiness (EDS) was measured using the Epworth Sleepiness Scale (ESS), a validated scale exploring eight everyday situations rated on a 4-point Likert scale ranging from “would never doze” to “high chance of dozing”. We consider a score of 16 or above as severe EDS [19]. This scale has shown great reliability and validity in a previous meta-analysis [20]. Fatigue was measured using a single item inspired by the Maslach Burnout Inventory (MBI): “Do you feel tired in connection with your work?” followed by a 7-point Likert scale from “Never” to “Daily” [21]. A frequency greater than once a week was considered to indicate significant fatigue. Anxiety and depressive symptoms were measured using the Patient Health Questionnaire 4 (PHQ-4), a short scale with 4 items rated on a 3-point Likert scale [22]. The first two items are summed to obtain the anxiety score and the last two to obtain the depressive score. We considered the participant to have significant “anxiety” or “depression” when the score was 3 or more on the specific scale.

Descriptive statistics were calculated as frequencies (%) for categorical variables, whereas means and standard deviations were computed for continuous variables. Univariate associations were presented using radar charts with the interquartile range as the scale. Multivariate associations were obtained using logistic regression to calculate the odds ratio (OR) and their 95% confidence interval (CI) between sleep–wake timings and significant mental complaints. Models were adjusted for age, gender, profession, financial situation and work schedules. In each model, we tested for interactions with exposure to COVID-19, finding no significant interaction. For all the tests, the accepted significance level was 5%. Data analyses were conducted using R v.3.4.3.

## 5. Conclusions

Healthcare workers are a specific population with a high prevalence of poor sleep hygiene and mental complaints. The COVID-19 crisis has worsened an already tense situation. The promotion of sleep health through behavioral sleep strategies should be encouraged to ensure good health for these professionals and good quality of care for their patients.

## Figures and Tables

**Table 1:** Descriptive characteristics (*n* = 1562).

Variables	*n* (%)	Mean ± SD {Min–Max}
Age - 18–30 - 30–45 - 45–60 - 60–75	1562 (100%) 358 (22.9%) 681 (43.6%) 494 (31.6%) 29 (1.9%)	40.0 ± 11.2 {18–75}
Gender - Female - Male	1562 (100%) 1258 (80.5%) 304 (19.5%)	
IMC - <18 - 18–25 - 25–30 - 30–40 - >40	1562 (100%) 36 (2.3%) 968 (62.0%) 377 (24.1%) 159 (10.2%) 22 (1.4%)	24.5 ± 6.0 {8–100}
Literacy - Basic scholarship - Bachelor’s degree - Master’s and PhD degrees	1562 (100%) 138 (8.8%) 1096 (70.2%) 328 (21.0%)	
Living environment - Rural - Urban	1562 (100%) 425 (27.2%) 1137 (72.8%)	
Work schedules - Fixed day - Shifted day - Night	1562 (100%) 840 (53.8%) 514 (32.9%) 208 (13.3%)	
Work in COVID-19 unit - Yes - No	1562 (100%) 265 (17.0%) 1297 (83.0%)	
Mean sleep duration (hours) - Less than 6 h - Between 6 and 7 h - More than 7 h	1562 (100%) 405 (25.9%) 484 (31.0%) 673 (43.1%)	6 h45 ± 54′ {5 h–9 h30}
Time-in-bed (hours) - Less than 6 h - Between 6 and 7 h - More than 7 h	1562 (100%) 93 (6.0%) 280 (17.9%) 1189 (76.1%)	7 h37 ± 54′ {5 h–14 h}
Sleep efficiency (%) - Less than 85% - Between 85% and 95% - More than 95%	1562 (100%) 379 (24.3%) 756 (48.4%) 427 (27.3%)	89% ± 10% {48%–100%}
Time-awake-in-bed - Less than 30 min - Between 30 and 60 min - More than 60 min	1562 (100%) 487 (33.9%) 460 (32.0%) 491 (34.1%)	52′ ± 46′ {0–8 h}
Sleep rebound - More than 2 h - Less than 2 h	1562 (100%) 427 (27.3%) 1135 (72.7%)	1 h36 ± 1 h26 {−6 h30–10 h}
Social jetlag - More than 2 h - Less than 2 h	1562 (100%) 179 (11.5%) 1383 (88.5%)	23′ ± 2 h38 {−11 h30–5 h30}
Insomnia (ISI) - Yes (ISI ≥ 15) - No (ISI ≤ 14)	1562 (100%) 530 (33.9%) 1032 (66.1%)	11.9 ± 5.6 {0–28}
Excessive daytime sleepiness (ESS) - Yes (ESS ≥ 11) - No (ESS ≤ 10)	1562 (100%) 704 (45.1%) 858 (54.9%)	10.1 ± 4.7 {0–24}
Sleep apnea (NOSAS) - Yes - No	1562 (100%) 142 (9.1%) 1420 (90.9%)	
Fatigue - Yes - No	1562 (100%) 205 (13.1%) 1357 (86.9%)	
Burnout - Yes - No	1562 (100%) 217 (13.9%) 1345 (86.1%)	
Anxiety (PHQ-4) - Yes - No	1562 (100%) 557 (35.7%) 1005 (64.3%)	2.4 ± 1.8 {0–6}
Depression (PHQ-4) - Yes - No	1562 (100%) 257 (16.5%) 1305 (83.6%)	1.4 ± 1.4 {0–6}

SD: Standard deviation.

**Table 2:** Multivariate associations between sleep hygiene and mental complaints (*n* = 1562).

Variable	Insomnia *p* OR [CI]	EDS *p* OR [CI]	Fatigue *p* OR [CI]	Depression *p* OR [CI]	Anxiety *p* OR [CI]
Mean sleep duration (hours) - More than 7 h - Between 6 and 7 h - Less than 6 h	***p* < 0.001** Ref **1.7 [1.3–2.2]** **3.8 [2.8–5.0]**	***p* = 0.031** Ref 1.1 [0.9–1.5] **1.4 [1.1–1.8]**	*p* = 0.646 Ref 1.2 [0.8–1.7] 1.1 [0.8–1.7]	***p* < 0.001** Ref **1.9 [1.3–2.6]** **1.7 [1.1–2.4]**	***p* < 0.001** Ref **2.0 [1.5–2.5]** **2.0 [1.5–2.6]**
Sleep efficiency (%) - Between 85% and 95% - Less than 85% - More than 95%	***p* < 0.001** Ref **1.9 [1.5–2.5]** 0.8 [0.6–1.1]	***p* = 0.026** Ref **1.4 [1.1–1.8]** 1.0 [0.8–1.3]	***p* = 0.003** Ref **1.9 [1.3–2.7]** 1.4 [0.9–2.0]	***p* = 0.022** Ref **1.5 [1.1–2.1]** 0.9 [0.6–1.3]	***p* = 0.004** Ref **1.5 [1.2–2.0]** 1.0 [0.8–1.3]
Sleep rebound - Less than 2 h - More than 2 h	***p* = 0.001** Ref **1.5 [1.2–2.0]**	*p* = 0.907 Ref 1.0 [0.8–1.3]	***p* = 0.026** Ref **1.5 [1.1–2.1]**	*p* = 0.557 Ref 1.1 [0.8–1.5]	*p* = 0.074 Ref 1.3 [1.0–1.6]
Social jetlag - Less than 2 h - More than 2 h	***p* < 0.001** Ref **1.9 [1.3–2.7]**	*p* = 0.254 Ref 0.8 [0.6–1.2]	*p* = 0.249 Ref 1.4 [0.8–2.2]	*p* = 0.726 Ref 0.9 [0.6–1.5]	*p* = 0.079 Ref 1.4 [1.0–2.0]

Significant associations in bold; adjusted for age, gender, profession, financial situation and work schedules.

## Data Availability

The raw data supporting the conclusions of this article will be made available by the authors on request.
